# Post-COVID-19 Longitudinally Extensive Transverse Myelitis with Myelin Oligodendrocyte Glycoprotein Antibodies

**DOI:** 10.1155/2022/1068227

**Published:** 2022-04-05

**Authors:** Ellen Yang, Adnan Husein, Jose Martinez-Perez, Terrence Li

**Affiliations:** ^1^Chicago Medical School, Rosalind Franklin University of Medicine and Science, North Chicago, IL, USA; ^2^Department of Clinical Sciences, Rosalind Franklin University of Medicine and Science, North Chicago, IL, USA

## Abstract

**Background:**

Myelin oligodendrocyte glycoprotein (MOG) antibody disease most commonly presents with optic neuritis, though myelitis is also possible. It is rare in the post-infectious and particularly post-COVID-19 setting. *Case Presentation*. We present the case of a 57-year-old man who tested positive for COVID-19 and experienced respiratory symptoms that completely resolved within one week. About 3 weeks after testing positive, he began experiencing acute onset anuria, followed by lower extremity paresthesia and paraparesis, which progressed to bilateral lower extremity paraplegia, complete loss of sensation of pain, temperature, vibration, and proprioception, and a T4 sensory level. He was initially diagnosed with and treated for acute inflammatory demyelinating polyradiculoneuropathy (AIDP), after which he made minimal clinical improvement. The diagnosis was shifted to longitudinally extensive transverse myelitis, and his CSF tested positive for MOG antibodies. He is being treated with a steroid regimen and extensive outpatient physical therapy.

**Conclusion:**

The neurologic manifestations of COVID-19 are still being uncovered. Neurologic symptoms should be included in patient education on symptom monitoring, even after recovery of respiratory illness, so that COVID-19-related CNS pathology can be urgently treated.

## 1. Introduction

The novel coronavirus caused a seismic disruption to healthcare establishments and scientific institutions around the globe, as they struggle not only to keep up with containing its spread and developing therapeutics, but also to gain an understanding of its postinfectious complications in patients, both immediate and long-term. It has been well established that viral infections can precipitate various postviral syndromes that affect the central nervous system (CNS). Coronaviruses, specifically HCoV-OC43 and SARS-CoV-1, have both been implicated in causing neurological complications [1]. There have been reports documenting post-COVID-19 transverse myelitis [2, 3] and acute disseminated encephalomyelopathy (ADEM) [4–7], [8], among others. We present the case of a man with a first-time diagnosis of myelin oligodendrocyte glycoprotein- (MOG-) associated longitudinally extensive transverse myelitis (LETM), likely postinfectious in etiology, secondary to COVID-19 infection. In our review of the literature, we have found only one other reported case of COVID-19-related new-onset MOG-associated myelitis [9], presented in the setting of concurrent infection. Literature review has yielded no other case reports of MOG-associated transverse myelitis in the context of post-COVID-19 infection.

## 2. Case Report

A 57-year-old right-handed male with recent COVID-19 three weeks prior to arrival presented to the ED with a 1-day history of acute onset, bilateral lower extremity numbness, paresthesia, paraparesis, and urinary retention. His medical history is unremarkable outside of essential hypertension, with no history of pulmonary, neurologic, or autoimmune disease. His lower extremity sensory and motor deficits progressively worsened over a 1-day period to paraplegia and complete loss of all sensory modalities in the lower extremities. He appeared ill and reported mild headaches, but denied fever, chills, shortness of breath, chest or abdominal pain, nausea, or vomiting.

Initial neurological examination revealed all lower extremity musculature unable to resist against gravity in both lower extremities. Deep tendon reflexes were normal and symmetrical. Decreased pinprick sensation (sensory level) was elicited bilaterally 1 fingerbreadth below the umbilicus. Upper extremity motor, sensation, and coordination examinations were normal. The Foley catheter was placed, and subsequent urinalysis showed no evidence of infection.

On admission, COVID-19 was detected via nasal swab antigen testing and confirmed via the Cepheid Xpert Xpress SARS-CoV-2 RT-PCR assay from a nasopharyngeal sample collected on 12/31/20. Patient was not complaining of respiratory symptoms, so lung imaging was deferred. MRI of cervical, thoracic, and lumbosacral spine with and without contrast showed no definitive spinal cord lesion. Neither CT nor MRI brain showed evidence of brain or brainstem lesions. Lumbar puncture (LP) was performed, and CSF showed slightly elevated protein (90 mg/dL), RBCs (40/1 mcL), and WBCs (9 cells/mm^3^), with neutrophilic predominance at 14%. LP was negative for oligoclonal bands, and an antinuclear antibody comprehensive panel, including SSA IgG, chromatin IgG, RPP IgG, SSB IgG, SM IgG, RNP IgG, JO 1 IgG, SCL70 IgG, centromere IgG, and SM/RNP, was negative. Labs were significant for serum glucose 109 mg/dL, otherwise unremarkable.

He was admitted with the working diagnosis of acute inflammatory demyelinating polyradiculoneuropathy (AIDP) versus postinfectious (COVID-19) radiculopathy. By the day after admission, the level of decreased pinprick sensation was elevated to the nipple line. The patient was started on intravenous immunoglobulin, but it was discontinued after 4 sessions due to development of subsegmental pulmonary embolus within right and left lower lobes, confirmed via CT chest pulmonary angiogram. He was then treated with 5 every other day sessions of plasmapheresis (PLEX). He was started on gabapentin for bothersome paresthesia.

Due to the presence of a spinal level and no improvement in his lower extremity sensorimotor deficits, repeat MRI cervical and thoracic spine was completed 15 days into admission.

**Figure 1:**
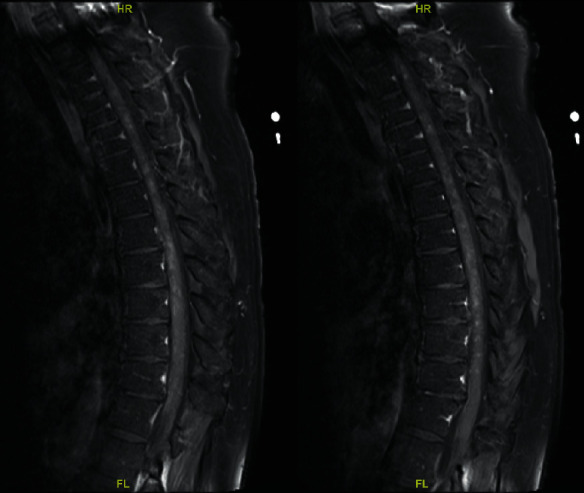
MRI spine T1-weighted sagittal images showing contrast enhancement.

**Figure 2:**
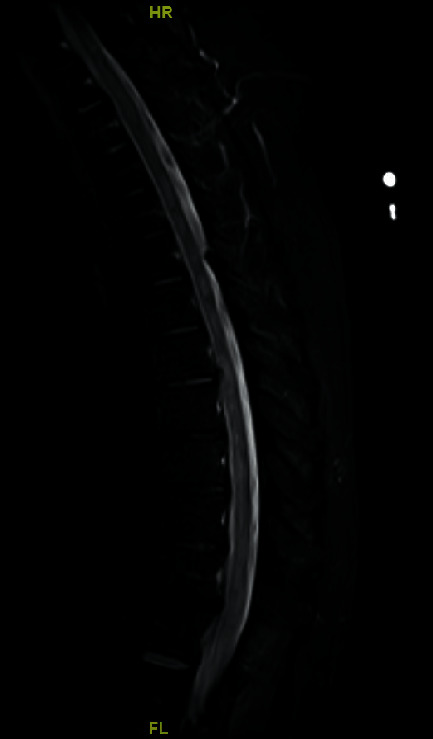
MRI spine T2-weighted sagittal image showing evidence of longitudinally extensive transverse myelitis (LETM).

Neuroimaging of the cervical spine demonstrated increased T2 signal throughout the spinal cord, with questionable enhancement of the left midline at C3. Thoracic spine imaging (Figures 1 and 2) showed multiple enhancing lesions along with spinal cord edema scattered throughout the length of the thoracic cord, consistent with longitudinally extensive transverse myelitis.

The patient was placed on a 5-day course of daily 1000 mg of IV solumedrol with slight improvement. Two weeks after, a serum autoimmune myelopathy panel sent to the Mayo Clinic was negative for amphiphysin antibody, AGNA-1, ANNA-2, ANNA-3, CRMP-5-IgG, DPPX antibody, GFAP, mGluR1, NIF, NMO/AQP4, N-type calcium channel antibody, P/Q-type calcium channel antibody, and PCA-1. The results were positive for anti-MOG IgG at 1 : 100 (Mayo Clinical Laboratories, Mayo, Rochester, MN) and glutamic acid decarboxylase (GAD65) antibody, indicative of CNS autoimmune disease. Serum interleukin-2 receptor (IL-2R) assay was positive at 1077 pg/mL, consistent with ongoing activation of the immune system, and serum Lyme IgG and IgM screens were negative, as was the PCR.

Given prior IV steroid treatment, our patient was placed on a reduced dose of 0.5 mg/kg daily prednisone starting at 40 mg to 20 mg to 10 mg to 5 mg, reducing the dose by half every month. A repeat LP was deferred as it would not have changed management for this patient.

## 3. Discussion

Transverse myelitis (TM) is an inflammatory condition of the spinal cord with a wide array of etiologies, including associations with autoimmune conditions, postinfectious, postvaccination, and idiopathic. Patients present with weakness, autonomic dysfunction, and a characteristic sensory level. Transverse myelitis with MRI lesions that span three or more vertebral levels are classified as having longitudinally extensive transverse myelitis [10]. LETM is often considered to be within the spectrum of NMO spectrum disorder (NMO-SD), which typically presents with bilateral optic neuritis, and has a strong association with aquaporin-4 (AQP4) antibodies (though not required), with MOG seropositivity being less common [11].

LETM does not always occur in the context of NMO and has been found to originate from various etiologies, with idiopathic and parainfectious being the next most common. Multiple sclerosis (MS) and acute disseminated encephalomyelitis (ADEM) have also been reported, as well as more rarely, myelin oligodendrocyte glycoprotein (MOG) antibody LETM [12].

With advances in antibody testing, MOG antibody disease (MOG-AD) is only relatively recently recognized as its own entity, separate from MS and NMO. MOG is a myelin surface glycoprotein found only in the CNS, with potential roles in mediating oligodendrocyte stability and complement mediation [13]. It is unclear whether the MOG antibodies themselves directly mediate the effects of the disease or if they are a coincidental accessory finding [13]. It is also been suggested that MOG antibodies have pathologic effects when the blood-brain barrier is compromised and provides the antibodies with access to the CNS [9]. The most common presenting symptom of MOG-AD is optic neuritis, followed by transverse myelitis like in our patient, and ADEM [14].

MOG-AD can occur spontaneously, but associations have been identified in the setting of post-Epstein-Barr virus and genital herpes simplex infections [15, 16]. In those cases, the MOG-AD manifested as ADEM and optic neuritis with meningoganglionitis, respectively. There has also been a reported pediatric case of post-Lyme disease MOG antibody-positive short segment myelitis and optic neuritis [17]. Postinfectious MOG antibody-positive LETM without optic neuritis appears to be a rare entity, with only two other documented cases reported in association with influenza A virus and varicella zoster virus [1, 18]. Vaccine-associated transverse myelitis is a well-described phenomenon, with reported associations including hepatitis B virus, measles-mumps-rubella (MMR), and diphtheria-tetanus-pertussis vaccines [19]. Postvaccination MOG-positive NMO-SD has been reported after MMR and varicella vaccines in a pregnant adult woman [20]. Interestingly, LETM has been reported following vaccination against COVID-19 with AZD1222, AstraZeneca [21].

Through the course of our patient's stay, there were multiple differential diagnoses considered. His presenting symptom of anuria, followed shortly by bilateral lower extremity paresthesia, paraparesis, and areflexia, all fit with a diagnosis of GBS, for which he was originally treated. The patient failed to make improvements on plasmapheresis and was later found to possess a sensory level on examination, shifting the working diagnosis towards TM. ADEM was considered, though the patient is lacking certain characteristic features, most notably, signs and symptoms of encephalopathy or any abnormal features on MRI of the brain.

Our patient never exhibited any signs or symptoms that were indicative of optic neuritis, and his clinical presentation indicated myelitis in the thoracic spine, supported by MRI findings. Compressive lesions were ruled out early on, and negative oligoclonal bands on CSF made MS less likely. Comprehensive workups including Lyme, ANA, GFAP, and more were negative. Finally, the positive GAD65 and MOG antibodies on the serum autoimmune myelopathy panel led us to our diagnosis of MOG antibody LETM. Of note, our patient's CSF was not tested for COVID, and therefore, primary CNS infection with COVID-19 cannot be ruled out. However, given the timeline of his disease course and absence of signs of CNS infection, we believe a postinfectious immune-mediated myelopathy to be more likely.

Characteristic MRI findings of the spinal cord can include signs of spinal cord inflammation, multifocal lesions, and spinal cord swelling. In a study of MOG antibody-positive individuals, 27/28 patients with a history of clinical myelitis showed spinal cord inflammation on MRI [14]. Both longitudinally extensive (greater than or equal to 3 continuous vertebral segments affected) and nonlongitudinally extensive (less than 3 continuous vertebral segments affected) are possible, sometimes concurrently [14]. LETM lesions in the study had a median length of 4 vertebral segments. Our patient had involvement throughout almost the entire thoracic spine.

Currently, no randomly controlled trials have been studied regarding MOG-AD treatment, likely due to its relative rarity and complexity. A retrospective case review found that documented treatments for MOG antibody-positive patients with acute attacks of optic neuritis and/or myelitis have included high-dose IV methylprednisolone (IVMP), plasma exchange, immunoadsorption, and oral steroids [14]. This study found that about 50% of patients were able to make full recoveries after steroid treatment.

Maciej et al. studied the prognosis of patients with MOG-AD and found that younger patients were more likely to fully recover than older patients [22]. Patients whose first presenting symptom was optic neuritis were more likely to relapse than those who presented with TM or ADEM. Furthermore, the majority of second-time attacks consisted of optic neuritis, even in patients whose initial presentation was TM. However, patients with TM as their initial presentation had significantly worse outcomes, including visual, motor, and autonomic dysfunction.

When comparing MOG and AQP4-associated NMO spectrum disorders, it has been found that NMO patients with MOG antibodies tended to have a single attack and/or fewer attacks than NMO patients with AQP4 antibodies who experienced significantly higher rates of relapsing disease [23]. Studies have also found that degree of recovery was different among the two groups, as determined based on the expanded disability status scale (EDSS); patients with AQP4 NMO scored significantly higher on the disability scale (more severe disability) than MOG NMO patients [11, 23].

## 4. Conclusion

MOG-AD can result in recurrent attacks of myelitis or optic neuritis, and sometimes, patients are left with permanent deficits. In cases of LETM, MOG antibodies should be included in the workup, especially in the context of AQP4 antibody negativity. As we continue to learn more about post-COVID-19 neurological complications, this case exemplifies the potential complexity of such cases. It highlights the importance of casting a wide differential and considering immune-mediated sequelae in diagnosing post-COVID-19 CNS disorders.

## References

[1] Amano H., Miyamoto N., Shimura H. (2014). Influenza-associated MOG antibody-positive longitudinally extensive transverse myelitis: a case report. *BMC Neurology*.

[2] Chakraborty U., Chandra A., Ray A. K., Biswas P. (2020). COVID-19-associated acute transverse myelitis: a rare entity. *BMJ Case Reports.*.

[3] Chow C. C. N., Magnussen J., Ip J., Su Y. (2020). Acute transverse myelitis in COVID-19 infection. *BMJ Case Reports*.

[4] Chen W. T., Huang Y. C., Peng M. C., Wang M. C., Lin K. P. (2016). Acute disseminated encephalomyelitis after influenza vaccination: a case report. *Critical Care Nurse*.

[5] Novi G., Rossi T., Pedemonte E. (2020). Acute disseminated encephalomyelitis after SARS-CoV-2 infection. *Neurol Neuroimmunol Neuroinflamm*.

[6] Parsons T., Banks S., Bae C., Gelber J., Alahmadi H., Tichauer M. (2020). COVID-19-associated acute disseminated encephalomyelitis (ADEM). *Journal of Neurology*.

[7] Lopes C. C. B., Brucki S. M. D., Passos Neto C. E. B. (2020). Acute Disseminated Encephalomye litis in COVID-19: presentation of two cases and review of the literature. *Arquivos de Neuro-psiquiatria*.

[8] Okhovat A. A., Ansari B., Hemasian H. (2020). Guillain-Barre syndrome in patients with coronavirus disease-2019: report of six cases and review of literature. *Current Journal of Neurology*.

[9] Zhou S., Jones-Lopez E. C., Soneji D. J., Azevedo C. J., Patel V. R. (2020). Myelin oligodendrocyte glycoprotein antibody-associated optic neuritis and myelitis in COVID-19. *Journal of Neuro-Ophthalmology*.

[10] Weinshenker B. G., Wingerchuk D. M., Vukusic S. (2006). Neuromyelitis optica IgG predicts relapse after longitudinally extensive transverse myelitis. *Annals of Neurology*.

[11] Kitley J., Waters P., Woodhall M. (2014). Neuromyelitis optica spectrum disorders with aquaporin-4 and myelin-oligodendrocyte glycoprotein antibodies: a comparative study. *JAMA Neurology*.

[12] Choudhary A., Bhargava A., Khichar S., Pradhan S. (2021). Etiological spectrum, clinico-radiological profile and treatment outcomes of longitudinally extensive transverse myelitis-a prospective study from Northwest India. *Journal of Neuroimmunology*.

[13] Wynford-Thomas R., Jacob A., Tomassini V. (2019). Neurological update: MOG antibody disease. *Journal of Neurology*.

[14] Jarius S., Ruprecht K., Kleiter I. (2016). MOG-IgG in NMO and related disorders: a multicenter study of 50 patients. Part 2: epidemiology, clinical presentation, radiological and laboratory features, treatment responses, and long-term outcome. *Journal of Neuroinflammation*.

[15] Nakamura Y., Nakajima H., Tani H. (2017). Anti-MOG antibody-positive ADEM following infectious mononucleosis due to a primary EBV infection: a case report. *BMC Neurology*.

[16] Nakamura M., Iwasaki Y., Takahashi T. (2017). A case of MOG antibody-positive bilateral optic neuritis and meningoganglionitis following a genital herpes simplex virus infection. *Multiple Sclerosis and Related Disorders*.

[17] Vieira J. P., Sequeira J., Brito M. J. (2017). Postinfectious anti-myelin oligodendrocyte glycoprotein antibody positive optic neuritis and myelitis. *Journal of Child Neurology*.

[18] Shiga Y., Kamimura T., Shimoe Y., Takahashi T., Kaneko K., Kuriyama M. (2017). *Rinsho Shinkeigaku*.

[19] Jurynczyk M., Messina S., Woodhall M. R. (2017). Clinical presentation and prognosis in MOG-antibody disease: a UK study. *Brain*.

[20] Sato D. K., Callegaro D., Lana-Peixoto M. A. (2014). Distinction between MOG antibody-positive and AQP4 antibody-positive NMO spectrum disorders. *Neurology*.

[21] Agmon-Levin N., Kivity S., Szyper-Kravitz M., Shoenfeld Y. (2009). Transverse myelitis and vaccines: a multi-analysis. *Lupus*.

[22] Kumar N., Graven K., Joseph N. I. (2020). *Case report:* postvaccination anti-myelin oligodendrocyte glycoprotein neuromyelitis optica spectrum disorder: a case report and literature review of postvaccination demyelination. *International Journal of MS Care*.

[23] Pagenkopf C., Südmeyer M. (2021). A case of longitudinally extensive transverse myelitis following vaccination against Covid-19. *Journal of Neuroimmunology*.

